# Birds as potential reservoirs of tick-borne pathogens: first evidence of bacteraemia with *Rickettsia helvetica*

**DOI:** 10.1186/1756-3305-7-128

**Published:** 2014-03-28

**Authors:** Sándor Hornok, Dávid Kováts, Tibor Csörgő, Marina L Meli, Enikő Gönczi, Zsófia Hadnagy, Nóra Takács, Róbert Farkas, Regina Hofmann-Lehmann

**Affiliations:** 1 Department of Parasitology and Zoology, Faculty of Veterinary Science, Szent István University, Budapest, Hungary; 2 Department of Evolutionary Zoology and Human Biology, Debrecen, Hungary; 3 Bird Ringing Station, Ócsa, Hungary; 4 Department of Anatomy, Cell and Developmental Biology, Eötvös Loránt University, Budapest, Hungary; 5 Clinical Laboratory and Center for Clinical Studies, Vetsuisse Faculty, University of Zurich, Zurich, Switzerland

**Keywords:** Ground feeding birds, Migratory birds, Ticks, *Rickettsia helvetica*, *Anaplasma phagocytophilum*, *Candidatus* Neoehrlichia mikurensis

## Abstract

**Background:**

Birds have long been known as carriers of ticks, but data from the literature are lacking on their role as a reservoir in the epidemiology of certain tick-borne disease-causing agents. Therefore, the aim of this study was to evaluate the presence of three emerging, zoonotic tick-borne pathogens in blood samples and ticks of birds and to assess the impact of feeding location preference and migration distance of bird species on their tick infestation.

**Methods:**

Blood samples and ticks of birds were analysed with TaqMan real-time PCRs and conventional PCR followed by sequencing.

**Results:**

During the spring and autumn bird migrations, 128 blood samples and 140 ticks (*Ixodes ricinus*, *Haemaphysalis concinna* and a *Hyalomma* specimen) were collected from birds belonging to 16 species. The prevalence of tick infestation and the presence of tick species were related to the feeding and migration habits of avian hosts. Birds were shown to be bacteraemic with *Rickettsia helvetica* and *Anaplasma phagocytophilum*, but not with *Candidatus* Neoehrlichia mikurensis. The prevalence of rickettsiae was high (51.4%) in ticks, suggesting that some of them may have acquired their infection from their avian host.

**Conclusion:**

Based on the present results birds are potential reservoirs of both *I. ricinus* transmitted zoonotic pathogens, *R. helvetica* and *A. phagocytophilum*, but their epidemiological role appears to be less important concerning the latter, at least in Central Europe.

## Background

Birds can fly over large distances in the course of a few days, particularly during their seasonal migration. Additionally, they have long been known for their epidemiological role as carriers of hard ticks (Acari: Ixodidae), implying that tick-borne pathogens in ticks attached to avian hosts can be transported to geographically distant places [1].

The majority of tick-borne pathogens are biologically transmitted, which means that these microorganisms infect their hard tick vector, where they multiply and/or develop prior to transmission to another vertebrate host [2]. On the other hand, ixodid larvae, nymphs and adult females suck blood on their host only once. Correspondingly, if larvae or nymphs acquire tick-borne pathogens with their blood meal, they will be able to infect another host only during a subsequent developmental stage, which is referred to as transstadial transmission [2]. Tick-borne pathogens taken up by adult female ticks will only be able to gain access to another host, if they pass through the ovaries/ova of the female ticks to the next generation (i.e. with transovarial transmission) [2].

Each species of tick-borne pathogen is able to infect a certain range of vertebrate host or reservoir species. The epidemiological significance of these hosts/reservoirs is that they can provide the source of infection for further tick vectors, thereby ensuring the maintenance of tick-borne pathogens in nature. Conversely, the tick-to-tick transmission of pathogens may not always necessitate the infection of the host/reservoir because tick-borne agents may spread between ticks feeding close to each other via the so-called co-feeding mechanism [3].

The most common tick species associated with avian hosts in various European countries is *Ixodes ricinus*[4-6]. According to the phenomenon of host-size restriction, usually the larvae and nymphs are the only stages of *I. ricinus* to suck blood on birds, whereas all three stages, i.e. larvae, nymphs and adults, feed on small, medium and large-sized mammals, respectively [7].

Compared to other tick species of the genus, *I. ricinus* is known as a competent vector of the highest number of important zoonotic bacteria [8]. Among these *Rickettsia helvetica* is considered an emerging tick-borne pathogen and is suspected to be involved in cases of human disease [9]. On the contrary, the majority of *I. ricinus*-borne pathogens may elicit clinical signs in both humans and animals, as exemplified by *Anaplasma phagocytophilum*, the causative agent of human, equine and canine granulocytic anaplasmosis, and of tick-borne fever in ruminants [10]. Recently, *I. ricinus* and its hosts were demonstrated to play an epidemiological role in the maintenance and transmission of *Candidatus* Neoehrlichia mikurensis, a newly described zoonotic pathogen [11]. In ticks, *Rickettsia* species have both transstadial and transovarial transmission [12], whereas members of the Anaplasmataceae family (including *A. phagocytophilum* and *Ca.* Neoehrlichia mikurensis) lack the latter [2].

In the above context, the aim of the present study was threefold: (1) to investigate whether the feeding preference and/or migration distance of different bird species influences their tick burden and thus their chances of becoming infected with certain tick-borne pathogens; (2) to evaluate whether birds can actually become infected/bacteraemic with three emerging, zoonotic tick-borne pathogens (*R. helvetica*, *A. phagocytophilum*, *Ca.* Neoehrlichia mikurensis); and (3) to compare the rate of infection with tick-borne pathogens among birds and their ticks, with relevance to transmission efficacy of these agents.

## Methods

### Sample collection

Birds were mist-netted during the spring and autumn migration periods of 2013 at the Ócsa Bird Ringing Station, Hungary (for six days in April, five days in May and two days in June, as well as for four days at the end of August and the beginning of September). The location and time periods were chosen by considering the migration stopover of birds in Hungary [13]. On the sampling days, 50 standard Ecotone mist-nets (Gdynia, Poland), 12 m in length, 2.5 m in height and with 16 × 16 mm holes, were used from 7:00 to 12:00 a.m. The whole body of each captured bird was scrutinized for the presence of ticks. All ticks were removed with fine forceps, and put into 70% ethanol in separate vials according to their hosts. Tick species were determined according to standard keys [7], and the feeding preference (ground level or above) and migration habit were assigned to bird species based on ornithological clues [13].

In addition, all tick carrier birds, as well as a similar number of tick-free individuals of the same bird species were blood sampled from the brachial vein using a fine (28G) needle and 0.5 ml syringe (Kendall Monoject: Tyco Healthcare Group Lp., Mansfield, MA, USA). Ticks were stored at room temperature, whereas blood samples were kept at -20°C.

### Ethical approval

The study was carried out according to the national animal welfare regulations (28/1998), and was approved by the Ethics Committee of the Faculty of Veterinary Science (SZIU).

### Sample preparation

Ticks were dried, washed three times (in detergent containing water, in tap water and in distilled water) and minced at the bottom of 1.5 ml Eppendorf tubes in 100 μl PBS with pointed scissors. Between each sample, the scissors were washed and flame sterilized for decontamination. Samples were then incubated overnight at 56°C in tissue lysis buffer containing proteinase-K. DNA was extracted from this lysate and from 20-100 μl of the blood samples using the QIAamp DNA Mini Kit (QIAGEN, Hilden, Germany) following the manufacturer’s instruction and using extraction control. The quantity and quality of bird blood DNA was assessed by PCR, which amplified part of the 18S rRNA gene [14]. These samples were consequently used at a dilution of 1:10.

### PCR methods

All PCR assays were performed using the appropriate positive and negative controls. The extraction controls were negative in all tests.

### *Candidatus* Neoehrlichia mikurensis

A multiplex real-time TaqMan PCR was applied to detect part of the 16S rRNA gene [15]. The assay is based on probes that indicate positivity on the family (Anaplasmataceae), genus (*Neoehrlichia*) and species (*Ca.* Neoehrlichia mikurensis) level.

### *Anaplasma phagocytophilum*

A TaqMan PCR was employed that amplifies part of the gene encoding a major surface protein (*msp2*) of *A. phagocytophilum*[16]. The probe was modified as 6-FAM-TGG TGC CAG GGT TGA GCT TGA GAT TG-TAMRA (5′-3′). The assay consisted of 40 cycles, and the results were considered positive if the threshold cycle (Ct) value was below 39.

### Rickettsiae

The presence of rickettsiae was evaluated by two TaqMan PCRs, detecting either part of the 23S rRNA gene of *R. helvetica* or part of the glt*A* gene for other rickettsiae [14]. The first test was specific for *R. helvetica*, and the Ct value was considered an inverse measure of the bacterial load [14]. In addition, to demonstrate the presence of *R. monacensis* in ticks, a conventional PCR amplifying a 381 bp long portion of the glt*A* gene [17] was performed, followed by gel electrophoresis (1.5% agarose) and sequencing of the positive samples (Macrogen Inc., Korea).

### Statistical analysis

Exact confidence intervals (CIs) for the prevalence rates at the 95% level were calculated according to Sterne’s method [18]. Sample prevalence data were analyzed using Fisher’s exact test. Differences were considered significant when P < 0.05.

## Results

### Tick infestation of birds

During the study, 1219 birds were captured. Tick infestations were found in 68 of these birds, belonging to 16 species (Table 1). Altogether 140 ticks were removed from the 68 birds. *Ixodes ricinus* was found on the majority of tick-infested birds (63 of the 68 individuals: 92.6%, CI: 83.7-97.6%). This was also the most abundant tick species (121 of the 140 ticks: 86.4%, CI: 79.6-91.6%) and was represented by 42 larvae, 78 nymphs and one adult female. Eight birds were infested with *Haemaphysalis concinna*, amounting to a prevalence of 11.8% (CI: 5.2-21.9%). The abundance of this species (with 13 larvae and five nymphs) was 12.6% (CI: 7.8-19.6%). Four birds were co-infested with *I. ricinus* and *Ha. concinna*. In addition, one Wood Warbler (*Phylloscopus sibilatrix*) carried a *Hyalomma* larva, which is not indigenous to Hungary.

**Table 1:** Prevalence and intensity of tick infestation among birds according to their feeding preference and migration habits

**Bird species (tick infested/all)**	**Feeding preference (tick infested/all)**	**Distance of migration (tick infested/all)**	**Number of birds infested with … (number of ticks)**				
			***Ixodes ricinus***		***Haemaphysalis concinna***		***Hyalomma *** **sp.**
			**Larva**	**Nymph**	**Larva**	**Nymph**	**Larva**
*Luscinia megarhynchos* (4/36)		**Long** (6/44)	2 (5)	4 (5)			
*Luscinia luscinia* (1/7)			1 (5)	1 (2)			
*Anthus trivialis* (1/1)			1 (1)	1 (4)			
*Erithacus rubecula* (13/52)	**Ground level** (27/115)		7 (9)	8 (14)			
*Turdus philomelos* (2/6)		**Short** (16/59)	1 (3)	2 (5)			
*Turdus iliacus* (1/1)				1 (1)			
*Turdus merula* (5/12)		**Local** (5/12)	2 (2)	4 (11)			
*Acrocephalus scirpaceus* (15/129)			2 (3)	12 (14)	2 (2)	3 (4)	
*Acrocephalus schoenobaenus* (4/90)			2 (2)	2 (2)			
*Locustella luscinioides* (1/26)		**Long** (23/283)			1 (5)		
*Sylvia communis* (2/19)			1 (1)	2 (2)			
*Phylloscopus sibilatrix* (1/19)	**Above ground level** (41/771)						1 (1)
*Sylvia atricapilla* (12/372)		**Middle** (13/464)	6 (10)	8 (8)	1 (1)	1 (1)	
*Phylloscopus collybita* (1/92)				1 (1)			
*Coccothraustes coccothraustes* (2/18)		**Short** (5/24)		2 (2)			
*Prunella modularis* (3/6)			1 (1)	3 (7)	1 (5)		

The prevalence of tick infestation was compared between two categories of bird species: those that preferentially feed from the ground and those that feed above ground level (Table 1). There were significantly (P < 0.0001) more tick-infested birds among those preferentially feeding from the ground, than among those feeding above the ground level, with a 23.5% (27 of 115, CI: 16.1-32.3%) and 5.3% (41 of 771, CI: 3.8-7.1%) prevalence of tick infestation, respectively. Neither *Ha. concinna* larvae nor nymphs were found on birds that feed on the ground (Table 1), indicating a significant difference in comparison with *I. ricinus* when considering either the prevalence (P = 0.018) or intensity of tick infestation (i.e. the total number of ticks per bird; P < 0.0001) (Table 1).

Considering bird species according to their migration characteristics (Table 1), the prevalence of tick infestation was 41.7% in the case of local bird species (5 of 12, CI: 15.2-72.3%) and 25.3% in the case of short distance migrants (21 of 83, CI: 16.4-36%). Both of these rates are significantly (P < 0.003) higher than those observed among middle and long distance migrants, with 2.8% (13 of 464, CI: 1.5-4.7%) and 8.9% (29 of 327, CI: 6-12.5%) prevalence of tick infestation, respectively. A *Hyalomma* larva was removed from a bird species with long distance migration (Table 1).

### Tick-borne pathogens in blood samples of birds

Blood samples were collected from all 68 tick-infested and 60 tick-free birds of the same species. Among the 128 blood-sampled birds six were found to be PCR positive with *R. helvetica* (4.7% prevalence, CI: 1.7-9.9%): these consisted of five Robins (*Erithacus rubecula*) and one Dunnock (*Prunella modularis*). The range of Ct values (33-40) reflected low to medium levels of bacterial loads. Another Dunnock harbored rickettsiae other than *R. helvetica* (the species could not be determined due to a high Ct value of 41). All rickettsia-positive birds were captured in April.

The blood sample of another bird (Redwing: *Turdus iliacus*), also caught in April, was PCR positive for *A. phagocytophilum* (Ct value 32.2). A further 25 birds carried unidentified member(s) of the Anaplasmataceae family (Ct values above 31.4), but none were positive for *Candidatus* Neoehrlichia mikurensis.

### Tick-borne pathogens in ticks collected from birds

Altogether, 72 of the 140 bird ticks (51.4%, CI: 42.8-60%) were PCR positive for rickettsiae: 61 harboured *R. helvetica* (43.6%, CI: 35.2-52.2%), and 11 contained *R. monacensis* (7.9%, CI: 4-13.6%). *R. helvetica* was found in both *I. ricinus* and *Ha. concinna* larvae and nymphs, but *R. monacensis* was only detected in *I. ricinus*. The proportions of rickettsia-positive *I. ricinus* larvae (22 of 42: 52.4%) and nymphs (42 of 78: 53.8%) were similar. Seven of the 18 *Ha. concinna* ticks (three larvae, four nymphs) carried *R. helvetica* (38.9%, CI: 17.3-64.3%).

Concerning the six *R. helvetica* PCR-positive birds, no ticks were found on four of them, and a PCR-negative tick was found on another. An additional *R. helvetica* bacteraemic bird carried 11 ticks, of which two (an *I. ricinus* nymph and a *Ha. concinna* larva) turned out to be PCR positive. At the same time, there were 38 *R. helvetica* PCR-negative birds, from which 59 *R. helvetica* PCR-positive ticks (including 17 *I. ricinus* and 2 *Ha. concinna* larvae) were collected.

In the *A. phagocytophilum*-specific PCR, only one *I. ricinus* nymph (removed from a PCR-negative bird) was positive. Conversely, the bird that tested positive for *A. phagocytophilum* carried a PCR-negative tick.

## Discussion

The prevalence of tick infestation among birds, as well as the species of ticks attaching to them, may depend on several factors that, in turn, influence the epidemiological role of avian reservoirs and hosts from the point of view of tick-borne diseases.

One of these factors is the feeding location preference of birds. In the present study, the prevalence of tick infestation was significantly associated with the habit of ground feeding in birds, similarly to previous observations [19]. However, the results shown here also revealed that this correlation may significantly depend on the tick species, as immature stages of *Ha. concinna* occurred exclusively on birds that preferentially feed above the ground. The difference noted herein between *Ha. concinna* and *I. ricinus* may be related to the host seeking behaviour of relevant immature stages. It has been reported that *Haemaphysalis* nymphs quest at various heights on vegetation according to the body size of the local deer population [20]. Similarly, in the present study, *Ha. concinna* larvae and nymphs infested birds that feed above ground level most likely because the preferred hosts of immature stages of this tick species in Hungary are roe deer [21]. Conversely, *I. ricinus* subadults apparently had lower questing heights (being highly prevalent on ground feeding birds) in association with rodents as primary hosts [22].

In the present study, it was also demonstrated that the distance of migration significantly influences the tick burdens of birds, concerning both the prevalence and the species composition. First, the prevalence of tick infestation was significantly higher among local birds and short distance migrants, most likely due to their presence/arrival during the main tick season, as suggested in other studies [4]. Bird species with middle to long distance migration habits are known to arrive in Hungary in May and June [13] (i.e. at the end or after the main tick season). On the other hand, a *Hyalomma* larva (which is not indigenous to Hungary) was removed from a long distance migrant captured in this study. In Europe, birds departing from southern locations to northern countries may carry Mediterranean hard tick species (e.g. two-host *Hyalomma* spp. that stay on the host as larvae and nymphs for 12-26 days [23]). Consequently, the epidemiological significance of birds with middle to longer distances of migration is the potential import of exotic tick species, as previously reported [6] and observed here.

The most significant result of the present study is the molecular evidence of bacteraemia in birds caused by *R. helvetica*. According to a previous report, when 130 wild birds were evaluated for the presence of rickettsiae, none were found to be rickettsaemic [24]. In another study 3% of 131 pooled bird blood samples were PCR positive for unknown rickettsiae [25]. Data are also available on the prolonged, experimentally elicited rickettsaemia of *R. rickettsii* and *R. canadensis* in birds [1,26]. Therefore, to the best of our knowledge, this is the first report of *R. helvetica* in the blood of any avian species. Both bird species with PCR-positive blood samples (the Robin and the Dunnock) are known for their synanthropic life (i.e. having urban and suburban habitats) [13].

Because there were no ticks on the majority of *R. helvetica* bacteraemic birds in the present study, and from one PCR-positive bird only a PCR-negative tick was collected, these data suggest rickettsaemia persists after the detachment of the vector tick in relevant avian hosts. At the same time, only two of the 11 ticks removed from a *R. helvetica* bacteraemic bird were PCR positive. Consequently, the transmission of rickettsiae from birds to tick vectors may occur with low efficacy. It is known that the bacterial load of *R. rickettsii* in the circulation of certain vertebrate reservoir hosts is enough to infect ticks, whereas rickettsaemia in other (also susceptible) hosts may be too low for this [27]. Alternatively, intermittent rickettsaemia was also reported in case of some *Rickettsia* spp. [28].

However, more than half (51.4%) of the ticks collected from birds in this study were found to be PCR positive for rickettsiae, mostly for *R. helvetica*. This prevalence is significantly higher than the infection rates reported in questing ticks collected from the vegetation (usually 4-16%, based on data from many countries: [9]). The prevalence of PCR positivity among *Ha. concinna* ticks from birds in this study (seven of 18 individuals) was also significantly (P < 0.001) higher, than the minimum and pool prevalence observed in the case of questing *Ha. concinna* ticks collected from the vegetation in Hungary (one of 53 pools were positive: [29]). The high prevalence of *R. helvetica* among immature ticks of birds in the present study may be explained by several potential causes. First, PCR positivity in larvae (especially on PCR-negative birds) can be partly attributed to the transovarial transmission of *R. helvetica*, as previously reported [30]. Second, the significantly higher prevalence among the immature stages reported here, when compared to adults in other studies, may indicate the low transstadial maintenance of *R. helvetica* (although the similar infection prevalence among larvae and nymphs as shown here, for both *I. ricinus* and *Ha. concinna*, argues against this). Third, some of the ticks may have acquired rickettsiae through co-feeding [1,3]. Last, but not least, some of the PCR-positive ticks collected from PCR-negative birds may even have acquired their *R. helvetica* infection from their avian host (PCR negativity of a bird at the time of blood sampling does not exclude rickettsaemia on other days of its tick’s blood sucking).

In addition to the birds positive for rickettsiae, there was one Redwing (*Turdus iliacus*) with detectable *A. phagocytophilum* bacteraemia. According to data in the literature, this may be the second piece of direct evidence for a natural avian infection or bacteraemia with this zoonotic pathogen, indicating that birds may play a role in the epidemiology of granulocytic anaplasmosis. In the initial study raising this possibility [10], the prevalence of *A. phagocytophilum* infection among birds was significantly higher (22%) than shown here, and, interestingly, it was the highest in Blackbirds (*Turdus merula*), which are taxonomically closely related to the species found to be PCR positive in the present study. On the other hand, data from the literature are contradictory when assessing the role of birds as reservoirs of *A. phagocytophilum* in comparison with small mammals. In one report, ticks removed from birds contained this pathogen with a significantly higher prevalence compared to those from the long known reservoirs (i.e. small mammals) [5]. However, another study (assessing the reservoir competence based on the molecular analysis of *A. phagocytophilum* in tick larvae from different mammalian and avian hosts) suggested a more important reservoir role of small mammals compared to birds [31].

There was only one *A. phagocytophilum*-positive tick on a bird that tested negative in the relevant PCR. The tick collected from the PCR-positive bird was also found to be negative for *A. phagocytophilum*. This is not surprising in light of the fact that the prevalence of *A. phagocytophilum* may frequently be very low in questing ticks (0.8%: [32]). This zoonotic pathogen was not even found in any of a large number of ticks collected from small mammals (known reservoirs) in Hungary [22].

Though *Candidatus* Neoehrlichia mikurensis was recently demonstrated in ticks of the same region where birds of this study were sampled [33], no PCR positivity was found for this zoonotic pathogen in birds. Nevertheless, this may be the first screening for *Ca.* Neoehrlichia mikurensis among birds (heavily infected with its vector *I. ricinus*). In the same multiplex PCR high numbers of bird blood samples were positive for member(s) of the Anaplasmataceae family. The species could not be further identified due to the low level of infection (high Ct values). This observation is in line with the presence of *Anaplasma* spp. in bird blood samples as reported previously [25].

## Conclusions

This is the first molecular evidence of *R. helvetica* infection in avian hosts. Based on the present results, birds are potential reservoirs of both *I. ricinus* transmitted zoonotic pathogens, *R. helvetica* and *A. phagocytophilum*, but their epidemiological role appears to be lower concerning the latter, at least in Central Europe. Our data also suggest that rickettsaemic birds may provide a source of infection for *I. ricinus* and *Ha. concinna*, but with low efficacy.

## Competing interests

The authors declare that they have no competing interests.

## Authors’ contributions

SH identified the ticks, extracted the DNA and wrote the manuscript. DK collected the blood samples and provided ornithological information. CST made possible bird capture and sample collection and supervised ornithological work. MM designed and, in part, performed the molecular analyses. ZSH collected the ticks. EG and NT performed most of the molecular analyses. RF supervised parasitological work. RH-L initiated and supervised the study. All authors read and approved the final version of the manuscript.
